# Bicarbonate-Rich Mineral Water Mitigates Hypoxia-Induced Osteoporosis in Mice via Gut Microbiota and Metabolic Pathway Regulation

**DOI:** 10.3390/nu17060998

**Published:** 2025-03-12

**Authors:** Yufan Ding, Weili Liu, Xi Zhang, Bin Xue, Xiaobo Yang, Chen Zhao, Chenyu Li, Shang Wang, Zhigang Qiu, Chao Li, Jingfeng Wang, Zhiqiang Shen

**Affiliations:** Military Medical Sciences Academy, Academy of Military Sciences, Tianjin 300050, China; dingyufan216@163.com (Y.D.); liuweili-002@163.com (W.L.); zhangxi0820@126.com (X.Z.); xue_bin04@163.com (B.X.); 18072712080@163.com (X.Y.); zhaochen212@126.com (C.Z.); nk_lcy710430@hotmail.com (C.L.); wsh847@163.com (S.W.); zhigangqiu99@gmail.com (Z.Q.); lc6628@163.com (C.L.)

**Keywords:** natural bicarbonate mineral water, high-altitude hypoxia, osteoporosis, gut microbiota, metabolism

## Abstract

**Background:** High-altitude hypoxia is known to adversely affect bone health, leading to accelerated bone loss and metabolic alterations. Recent studies suggest that factors such as bicarbonate and gut microbiota may play key roles in bone health. Mineral water, rich in bicarbonate, may influence bone health and the gut–bone axis under such conditions. **Methods:** Mice were exposed to hypoxia and treated with different concentrations of drinking water. Bone-related parameters were assessed using dual-energy X-ray absorptiometry (DXA) and Micro-CT. Bone health was assessed using the measurement of serum biomarkers. Additionally, Untargeted Metabolomics was employed to analyze differential metabolites between groups, while gut microbiota composition was analyzed using 16S rRNA sequencing. **Results:** BMW consumption increased bone mineral density (BMD) and helped alleviate the damage to the microstructure of bones caused by hypoxia and delayed the progression of osteoporosis. Additionally, BMW was shown to enhance probiotics such as *Akkermansia* and *Dubosiella* and regulate the longevity-regulating pathway as well as the PI3K/AKT/mTOR (PAM) signaling pathway. This study also discovered changes in metabolic products due to BMW intervention, predominantly in pathways such as the amino acid, prostaglandin, and purine metabolisms, with correlation analysis further exploring the relationships between gut microbiota and these differential metabolites. **Conclusions:** Long-term exposure to high-altitude hypoxic conditions affects the structure of gut microbiota and bone metabolism in mice. The consumption of BMW improves the structure of gut microbiota and regulates the metabolic pathways to maintain bone health under high-altitude hypoxia.

## 1. Introduction

Water is one of the most essential nutrients for daily life, with minerals in water typically existing in ionic form, which enhances their absorption in the intestinal tract. The World Health Organization (WHO) has highlighted that minerals in drinking water can partially supplement the population’s intake of essential nutrients [1]. However, much of the current research on drinking water focuses on the health effects of mineral cations [2,3,4], with less emphasis on the benefits of anions. Among these, bicarbonate—the most abundant inorganic salt in freshwater—is a critical component of the human body’s buffering system [5]. Notably, serum bicarbonate levels have been significantly correlated with bone mineral density (BMD), suggesting that serum bicarbonate should be included in the diagnostic assessment of osteoporosis [6]. Clinical attempts to improve osteoporosis through oral bicarbonate therapy (sodium bicarbonate, potassium bicarbonate) have shown limited results and scope of use, indicating a need for long-term experiments to evaluate its effects and safety [7,8]. This opens the door for exploring bicarbonate’s role in the prevention and management of osteoporosis, especially in populations at risk. Osteoporosis is a systemic bone disease characterized by low bone mineral density (BMD) and damage to the microstructure of bone tissue [9]. It arises from an imbalance between bone formation and resorption, where excessive resorption leads to a reduction in bone mineral content and decreased BMD [10]. As a chronic metabolic disease, osteoporosis is increasingly prevalent globally, with factors such as population aging, menopause in women, and deficiencies in calcium and vitamin D now recognized as significant risks [11,12,13]. Moreover, environmental factors, including high-altitude hypoxia, are critical contributors to the development of osteoporosis. High-altitude areas, characterized by lower oxygen levels, cold temperatures, and increased radiation, can disrupt bone homeostasis by altering the balance between bone formation and resorption [14], leading to the over-proliferation of osteoclasts and an increase in their size [15,16], which accelerates bone density loss. Additionally, hypoxia impairs the osteogenic potential of mesenchymal stem cells (MSCs) and inhibits osteoblast differentiation, further exacerbating osteoporosis [17]. Epidemiological studies have shown that the prevalence of osteoporosis is significantly higher in high-altitude regions compared to lowland areas [18].

Emerging evidence also suggests that gut microbiota play a pivotal role in various physiological processes, including digestion, nutrient absorption, metabolism, immune regulation, and vitamin production [19]. Notably, external environmental factors can influence the gut microbiota, with high-altitude hypoxia being linked to gastrointestinal dysfunction and changes in microbiota composition [20,21]. Recent studies have demonstrated a strong connection between gut microecology and bone health [22,23], suggesting that the gut microbiota may be a potential target for the prevention and treatment of osteoporosis. The current treatment of osteoporosis primarily relies on medication, with nutrition supplementation serving as an adjunct. However, clinical treatment still faces numerous limitations, making early prevention particularly crucial for osteoporosis at high altitudes.

Given these challenges, drinking water interventions present an easily implementable and sustainable preventive strategy. Recent research has highlighted the influence of alkaline water on gut microbiota composition [24] and the potential effects of gut-derived metabolites on bone health [23,25,26]. However, the role of natural bicarbonate mineral water (BMW) in improving bone metabolic homeostasis, particularly in the context of hypoxia and along the gut–bone axis, remains poorly understood.

In this study, we explore the potential benefits of long-term BMW consumption in mice exposed to high-altitude hypoxia. Our objective was to investigate whether BMW can improve bone metabolism and homeostasis in hypoxic conditions. To elucidate the underlying mechanisms, we employed 16S rRNA sequencing and Untargeted Metabolomics to assess the effects of BMW on gut microbiota composition, systemic metabolism, and bone health.

## 2. Materials and Methods

### 2.1. Drinking Water for Experiment

Four different types of drinking water were selected for this experimental intervention. Tap water (TP) was sourced from Tianjin Water Group Co., Ltd. (Tianjin, China), and adhered to the Chinese National Drinking Water Standard GB 5749-2022 [27], which sets nationwide quality requirements for tap water. Purified water (PW) refers to commercially available bottled water with low mineral content (Wahaha Pure Water, Hangzhou Wahaha Group Co., Ltd., Hangzhou, China). Natural bicarbonate mineral water (BMW) is a commercially available bottled water with high calcium and bicarbonate content (Gerolsteiner Mineralwasser, Gerolsteiner Brunnen GmbH & Co. KG, Gerolstein, Germany). Regular mineral water (RMW) is produced by neutralizing the bicarbonate in BMW with sulfuric acid while maintaining similar levels of key cations, such as calcium and magnesium, to those in BMW. Throughout the experiment, commercially available water from the same production batch was used, with periodic checks on mineral concentrations being performed to ensure the stability of water quality.

### 2.2. Animals and Experimental Groups

A total of 60, 7-week-old, male C57BL/6 Nifdc mice were purchased from Beijing Vital River Laboratory Animal Technology Co., Ltd. (Beijing, China), to be experiment animals. Mice were housed under Specific Pathogen-Free (SPF) systems, with a 12 h light/12 h dark cycle (lighting phase from 06:00 to 18:00 daily) in a temperature-controlled room maintained at 23 ± 2 °C and a relative humidity of 45–55%. The cages were furnished with appropriate bedding material (e.g., wood shavings), and the bedding was changed regularly to maintain a clean environment. Mice had free access to food (Laboratory animals—Nutrients for formula feeds, GB 14924.3-2010 [28]) and water in their cages and were habituated to the conditions for 7 days prior to the experiment procedure. During the course of the experiment, any signs of distress or illness were carefully monitored, and appropriate veterinary care was provided as needed. All experimental protocols were approved by the institutional animal care and use committee (IACUC) and adhered to the ethical guidelines for animal welfare.

**Figure 1:**
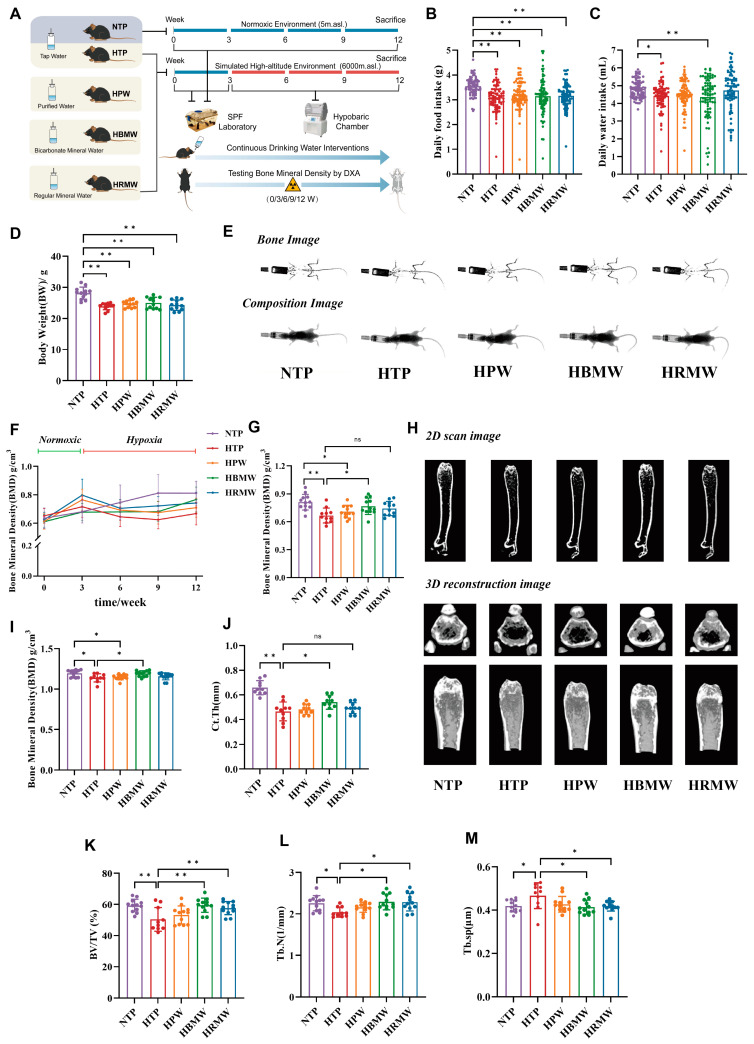
Recording of basic information on mice, imaging results, and statistical analysis. (**A**) Experimental scheme of different drinking water on high-altitude hypoxia-induced osteoporosis. (**B**–**D**) Statistical analysis of daily food, water intake, and bodyweight in mice. (**B**) Food intake. (**C**) Water consumption. (**D**) Body weight. (**E**–**G**) Bone densitometry and indexing in mice using DXA. (**E**) Body composition imaging of mice using DXA. (**F**) Periodic BMD of mice. (**G**) Femoral BMD in Week 12. (**H**–**L**) Test of ex vivo femur of mice using Micro-CT. (**H**) 2D scan images and 3D reconstruction of femur (horizontal reconstruction in the top row, vertical in the bottom row). (**I**) BMD, (**J**) Ct.Th, (**K**) BV/TV, (**L**) Tb.N, and (**M**) Tb.sp. ns: no significance, * *p* < 0.05, ** *p* < 0.01.

Mice were randomly divided into 5 groups: normoxic tap water (NTP, *n* = 12), hypoxia tap water (HTP, *n* = 12), hypoxia purified water (HPW, *n* = 12), hypoxia bicarbonate mineral water (HBMW, *n* = 12), and hypoxia regular mineral water (HRMW, *n* = 12). All of them were first kept in a normoxic environment at an altitude of 5 m (Tianjin, China) and given different drinking water interventions for three weeks to develop water intake habits. The food and water intake of mice were measured every day, the body weight was assessed every week, and, after 3 weeks, the NTP continued to place at normoxic conditions, while the four hypoxia groups were housed in a controlled hypobaric chamber simulating 6000 m above sea level. The bone density (BMD) of mice was examined using dual-energy X-ray absorptiometry (DXA) every three weeks. After 9 weeks of continuous exposure, all animals were euthanized. Blood was immediately collected for biochemical analysis and stored at −80 °C until use. Femurs were isolated for imaging, histology, and mechanics-related tests. The microbiota in mouse feces was analyzed using 16S rRNA sequencing, and metabolites were analyzed using Untargeted Metabolomics (Figure 1A).

### 2.3. Hypobaric and Hypoxic Exposure

Hypoxia groups were placed in a low-pressure oxygen chamber (ProOx-810, TOW TECH, Beijing, China), simulated altitude of 6000 m, and light and dark circulation was carried out at 12 h: 12 h and 20 h/d for chronic long-term hypobaric hypoxia intervention for 9 w. Daily hypoxia was interrupted for 4 h in order to carry out a daily measurement of food and water intake and cleaning of cages, and body mass was weighed periodically.

### 2.4. Mouse Bone Densitometry

The mice were anesthetized by inhalation of isoflurane using a small-animal anesthesia machine (Matrx VMR, DHS BIOTECH, Tianjin, China). After the mice became soft and immobile, the mice were transferred to the bone densitometer (InAlyzer, MEDIKOR, Seongnam, Republic of Korea), and the gas anesthetics were continuously inhaled through the nose in order to keep the mice in a continuous anesthetic state. The mice were numbered according to their grouping, and their weight, age, and sex were entered into the BMD scanner. After entering the Result interface, the ROI tool was used to circle the left and right femur morphologies, and the built-in BMD software was used to calculate the bone density of the ROI area of the mice and record the data.

### 2.5. Micro-CT Scanning and 3D Reconstruction

The mice ankle joint was placed in the scanning table of a micro-CT animal imager (Quantum FX; PerkinElmer, Waltham, MA, USA), and the imaging parameters were set as follows: voltage of 90 kV, current of 180 μA, FOV of 40 mm, and 360° scanning 4.5 min. After scanning, the VOL EDIT module in Caliper analyze software was used to reconstruct the region of interest of the sample in three dimensions. After the three-dimensional reconstruction was completed, it was imported into the Measure module of Caliper analyze to calculate the morphological parameters of the trabecular bone, such as bone volume fraction (BV/TV, %), trabecular thickness (Tb.Th, μm), trabecular number (Tb.N, 1/mm), trabecular bone spacing (Tb.Sp, μm), structure model index (SMI), and the mean bone mineral density (BMD).

### 2.6. Three-Point Bending Test

An electronic universal testing machine (EUTM) with a load range of 0.00001 N and a beam displacement resolution of 0.00001 mm was used. Mouse femurs were used as the study object, in which the span (*L*, mm) was 10 mm and the loading speed was 1 mm/min. The maximum load (*F_max_*) and maximum deflection (*d_max_*) were tested and recorded using the electronic universal testing machine. The maximum (*B*) and minimum (*H*) values of the outer diameter of the cross-section were measured by taking the broken femur after the test.

(1) Cross-sectional moment of inertia

When the specimen is loaded to its maximum and fracture occurs, the moment of inertia of the cross-section (*J*, mm^2^) is calculated as follows:

$$J={\pi BH}^{3}/64$$

(2) Maximum bending stress:

$$\sigma_{b}=F_{max}LH/8J$$

(3) Maximum strain:

$$\varepsilon_{b}=12d_{max}H/2L^{2}$$

### 2.7. Preparation and Staining of Mouse Femur Sections

Bone specimens were decalcified in EDTA. After confirming that the bone tissue specimens were sufficiently softened, gradient dehydration was performed using different concentrations of ethanol. Bone tissue specimens were immersed in wax for embedding. The wax blocks were trimmed at −20 °C on a freezer table to ensure adequate cooling. Sections were made in a slicer to a thickness of approximately 4 μm. Then, 40 °C warm water was used to flatten the slices, and slides were used to retrieve the slices, removed from the oven after drying in water and baking the wax, and reserved for later use.

Paraffin sections were dewaxed and washed. The sections were stained with hematoxylin staining solution for 3–5 min, washed, differentiated, washed, returned to blue, and washed. The sections were gradient dehydrated with different concentrations of ethanol and stained with eosin stain for 5 min. the sections were removed after gradient dehydration until they were transparent, and the sections were sealed with neutral gum.

### 2.8. Routine Blood Tests and Analysis of Serum Indicators

Blood sampling from the tail vein of mice was carried out at the end of the experiment and transferred into test tubes containing EDTA. Blood cell counts were measured within 4 h using a Veterinary Automatic Blood Cell Analyzer (BC-5000vet, Mindray Animal Care Technology Co., Shenzhen, China). Serum total calcium levels were measured using an Automatic Animal Biochemical Analyzer (BS-240vet, Mindray Animal Care Technology Co., Shenzhen, China). Cross-linked C-Telopeptide of type I collagen (CTX I, F43275-A, ELISA method), bone alkaline phosphatase (BALP, F2775-A, ELISA method), propeptide of type I procollagen (PINP, F30285-A, ELISA method), parathyroid hormone (PTH, F2536-A, ELISA method), bone gla-protein (BGP, F2551-A, ELISA method), calcitonin (CT, F2562-A, ELISA method), and 25-hydroxyvitamin D(25-OH-VD, F30413-A, ELISA method) in the serum were analyzed using commercial assay kits (FANKEW Co., Ltd., Shanghai, China). Detailed operation procedures were performed following the manufacturer’s product manual.

### 2.9. 16S rRNA Sequencing

Genomic DNA was extracted using the Cetyltrimethylammonium Ammonium Bromide (CTAB) method, with sequencing sequences selected from primers in the 16S V4 region, Phusion^®^High-Fidelity PCR Master Mix with GC Buffer (New England Biolabs, Beijing, China) to operate the PCR to ensure highly accurate amplification rates. The PCR products were purified using magnetic beads, mixed in equal amounts according to the concentration of the PCR products, and, after sufficient mixing, the PCR products were detected and the target bands were recovered for library construction, the constructed libraries were quantified using Qubit and Q-PCR, and, after the libraries were qualified, the NovaSeq6000 (Illumina, San Diego, CA, USA) was used to perform the on-board sequencing of the PE250.

Barcode and primer sequences were truncated and spliced using FLASH [29] (Version 1.2.11, http://ccb.jhu.edu/software/FLASH/, accessed on 2 March 2024) for each sample of reads, and the resulting spliced sequences were the Raw Tags. The Raw Tags were strictly filtered using fastp software (Version 0.23.1) to obtain high-quality Tags (Clean Tags) [30], which were compared with reference databases (Silva database https://www.arb-silva.de/, accessed on 6 March 2024) for 16S/18S, Unite database https://unite.ut.ee/, accessed on 10 April 2024) for ITS, and the chimeric sequences were removed to obtain the Effective Tags. We used the DADA2 module in QIIME2 (Version QIIME2-202202) for noise reduction to obtain the final ASVs (Amplicon Sequence Variants). The ASVs were annotated with species based on the clustering results to obtain information about the species and their distribution. And then species diversity, abundance structure, and differential species analyses among different samples were performed using bioinformatics methods.

### 2.10. Untargeted Metabolomics

Feces (100 mg) were individually grounded with liquid nitrogen, and the homogenate was resuspended with prechilled 80% methanol using a well vortex. The samples were incubated on ice for 5 min and then were centrifuged at 15,000× *g*, 4 °C, for 20 min. Some of the supernatant was diluted to the final concentration containing 53% methanol using LC-MS grade water. The samples were subsequently transferred to a fresh Eppendorf tube and were centrifuged at 15,000× *g*, 4 °C, for 20 min. Finally, the supernatant was injected into the LC-MS/MS system analysis [31,32].

UHPLC-MS/MS analyses were performed using a Vanquish UHPLC system (Thermo Fisher, Dreieich, Germany) coupled with an Orbitrap Q ExactiveTM HF mass spectrometer or Orbitrap Q ExactiveTMHF-X mass spectrometer (Thermo Fisher, Dreieich, Germany) in Novogene Co., Ltd. (Beijing, China). Samples were injected onto a Hypersil Goldcolumn (100 × 2.1 mm, 1.9 μm) using a 12 min linear gradient at a flow rate of 0.2 mL/min. The eluents for the positive and negative polarity modes were eluent A (0.1% FA in Water) and eluent B (Methanol). The solvent gradient was set as follows: 2% B, 1.5 min; 2–85% B, 3 min; 85–100% B, 10 min; 100–2% B, 10.1 min; 2% B, 12 min. Q ExactiveTM HF mass spectrometer was operated in positive/negative polarity mode with spray voltage of 3.5 kV, capillary temperature of 320 °C, sheath gas flow rate of 35 psi, and Aux gas flow rate of 10 L/min, S-lens RF level of 60, and Aux gas heater temperature of 350 °C.

The raw data files generated using UHPLC-MS/MS were processed using Compound Discoverer 3.3 (CD3.3, Thermo Fisher, Waltham, MA, USA) to perform peak alignment, peak picking, and quantitation for each metabolite. The main parameters were set as follows: peak area was corrected with the first QC, actual mass tolerance, 5 ppm; signal intensity tolerance, 30%; and minimum intensity et al. After that, peak intensities were normalized to the total spectral intensity. The normalized data were used to predict the molecular formula based on additive ions, molecular ion peaks, and fragment ions. And then peaks were matched with the mzCloud (https://www.mzcloud.org/, accessed on 4 June 2024), mzVault, and MassList databases to obtain the accurate qualitative and relative quantitative results. These metabolites were annotated using the KEGG database (https://www.genome.jp/kegg/pathway.html, accessed on 11 June 2024), HMDB database (https://hmdb.ca/metabolites, accessed on 13 June 2024), and LIPIDMaps database (http://www.lipidmaps.org/, accessed on 25 July 2024).

### 2.11. Statistical Analysis

Statistical analysis was performed using SPSS 27.0 statistical software. The figures were prepared using GraphPad Prism 9 software. The data were expressed as Mean ± SD. A one-Way ANOVA test was used to compare the means among multiple groups, and an LSD test was used for multiple comparisons after the event. When the test result was *p* < 0.05, the difference between groups was considered significant. Alpha diversity was determined by Simpson and Shannon’s test, and the significance of differences was verified by Kruskal–Wallis test and Wilcoxon test; Beta diversity was visualized using PCA, PCoA, and permutation multivariate analysis of variance (PERMANOVA) to assess differences in beta-diversity. OPLS-DA VIP > 1 and *p* < 0.05 were the screening criteria for significantly different metabolites. Correlations between microbiota and metabolite data were calculated using the Spearman algorithm.

## 3. Results

### 3.1. Minerals in Water

Four types of drinking water were selected for this study. Tap water (TP) was used as a control. Purified water (PW) is characterized by very low mineral and bicarbonate (BIC) content. Bicarbonate mineral water (BMW) has a high content of BIC, Ca, and Mg, while regular mineral water (RMW) is based on BMW but uses sulfuric acid to neutralize BIC. It features similar mineral ion concentrations to BMW, but with significantly reduced BIC levels and increased sulfate content (Table 1).

### 3.2. Bodyweight, Diet, and Water Consumption

Mice in the normoxic group (NTP) were in good condition throughout the experimental period. In contrast, other groups in the hypoxic environment showed characteristic hypoxic manifestations such as decreased vigor, curling up, withered fur, dark red paws, tail, and ears, as well as purplish lips and tongues to varying degrees. Especially during the first week of exposure to high-altitude hypoxia, food and water intake decreased rapidly, but gradually increased after a week of acclimatization. The groups showed significant differences in body weight, diet, and water intake at the end of the experiment (Figure 1B–D). A statistical analysis of the amount of food and water consumed during the entire experimental period revealed that the amount of food and water consumed, as well as the body weight of HTP group, were significantly lower than those of the NTP. However, the amount of food and water consumed and the body weight among the four different water groups were not significantly different.

### 3.3. Protective Effect of BMW Against High-Altitude Hypoxia-Induced Osteoporosis

#### 3.3.1. BMW Maintains Bone Mineral Density Under High Altitude Hypoxia Exposure

After anesthetizing the mice with isoflurane, the bones of the mice were photographed in vivo using the DXA method, and their body composition was analyzed (Figure 1E). Statistical analysis of the total BMD revealed high variability between the values of the groups, and the measurement of BMD in the femoral region was calculated using the ROI tool circling the femoral morphology, which showed high stability (Supplementary Figure S1). Therefore, subsequent scanning measurements were performed every three weeks using mouse femur BMD as an observational indicator.

The results show that, during the normoxic environment exposure period (0–3 weeks), all mice showed an increasing trend in BMD. At the early stage of hypoxia exposure (3–6 weeks), the BMD of the HTP, HPW, and HRMW groups decreased significantly compared to the previous stage, while no decreasing trend was observed in the HBMW group. At the middle stage of the subsequent hypoxia intervention (6–9 weeks), the BMD of HBMW remained relatively stable, while the decreasing trend persisted in the HTP and HPW groups. There was a tendency for the HRMW group to rebound compared to the previous stage. By the end of the hypoxia exposure (9–12 weeks), BMD showed an increasing trend in all four hypoxia groups, with the HBMW group demonstrating the highest rate of increase (Figure 1F). We analyzed the BMD obtained at the end of the experiment (Figure 1G). The BMD value of the HTP group was significantly lower than that of the NTP, suggesting that high-altitude hypoxia-induced osteoporosis modeling was successful. The BMD of the HBMW group was significantly higher than that of the HTP group, suggesting that long-term consumption of BMW could maintain BMD under high-altitude hypoxic conditions.

Then mice were sacrificed and femurs were taken for micro-CT and three-dimensional (3D) reconstruction and bone-related parameterization (Figure 1H–M). The results show that, after hypoxic exposure, the number of trabeculae in the cross and longitudinal sections of the mouse femur decreased and became sparse, the thickness of cortical bone decreased, and the bone tissue structure was severely disrupted. Bone parameter measurements indicate that, in the mouse femur, BMD, bone volume fraction (BV/TV), trabecular number (Tb.N), and cortical bone thickness (Ct.Th) were significantly lower, while trabecular separation (Tb.sp) markedly showed a higher value. Overall, these findings suggest that long-term exposure to high-altitude hypoxia induces osteoporosis. After different water interventions, compared to the HTP group, mice in the HBMW group showed higher and densely arranged trabeculae in both the cross-sectional and longitudinal views of the femur, thicker cortical bone, and a gradual normalization of bone tissue structure. The HRMW group also exhibited some improvement in bone microstructure. The bone parameter analysis revealed that drinking mineral water (HBMW and HRMW) increased BV/TV and Tb.N under high-altitude exposure and reduced Tb.sp. However, only the HBMW group effectively prevented the decrease in BMD and Ct.Th and significantly alleviated the progression of osteoporosis.

#### 3.3.2. BMW Improves Bone Resorption and Formation Related Indicators Under High-Altitude Hypoxia Exposure

**Figure 2:**
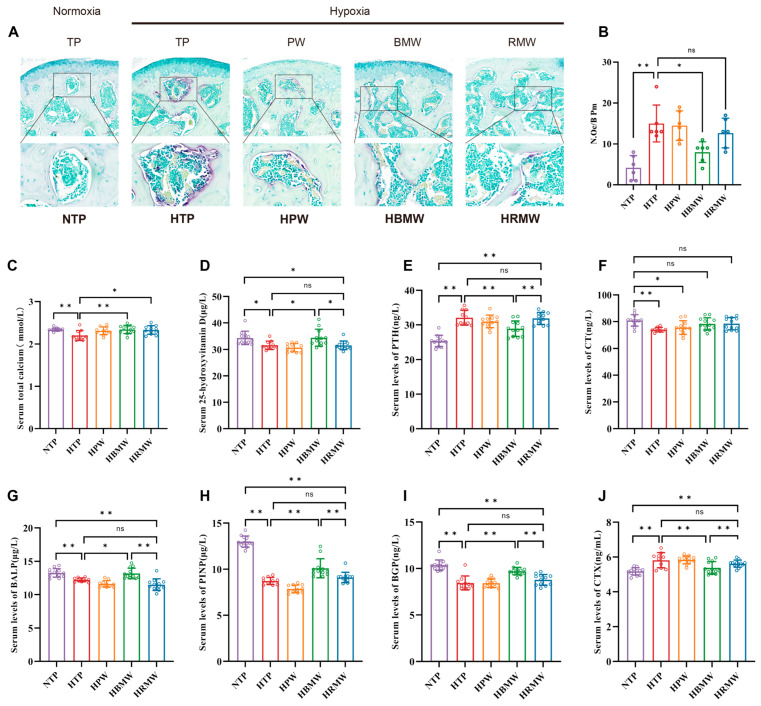
Evaluation of indexes related to bone resorption and bone formation in mice. (**A**,**B**) TRAP staining and counting of osteoclasts. (**A**) Microscopic observation of femur TRAP staining (40×). (**B**) Number of osteoclasts per field of view. (**C**–**F**) Serum indicators of calcium and phosphorus metabolism. (**C**) Serum total calcium, (**D**) 25-OH-VD, (**E**) PTH, and (**F**) CT. (**G**–**I**) Serological indicators of bone formation, (**G**) BALP, (**H**) PINP, and (**I**) BGP. (**J**) Serological indicators of bone resorption, CTX. ns: no significance, * *p* < 0.05, ** *p* < 0.01.

We performed TRAP staining on mouse femurs. The results show a significant higher value in purple-stained osteoclasts in the HTP and HPW groups following exposure to high-altitude hypoxia. Conversely, purple-stained osteoclasts were significantly reduced in the HBMW group (Figure 2A,B). Then, we measured the bio-markers associated with bone resorption and formation in serum (Figure 2C–J). Disturbances in the calcium and phosphorus metabolism were found in mice after long-term chronic hypoxia, with lower values in serum total calcium, serum 25-hydroxyvitamin D (25-OH-VD), calcitonin (CT), and higher values in parathyroid hormone (PTH). In contrast, the values of serum total calcium, 25-OH-VD, and PTH were significantly attenuated in the HBMW group compared to the HTP group. Exposure to high-altitude hypoxia also resulted in lower values in bone formation bio-markers, including bone alkaline phosphatase (BALP), propeptide of type I procollagen (PINP), and bone gla-protein (BGP) in mice, while the bone resorption bio-marker cross-linked with C-telopeptide of type I collagen (CTX) was higher, leading to the occurrence of osteoporosis. All of the above indicators improved in the HBMW group, but no significant difference was observed in the HRMW group.

#### 3.3.3. BMW Improves Bone Support and Hematopoiesis Under High Altitude Hypoxia Exposure

**Figure 3:**
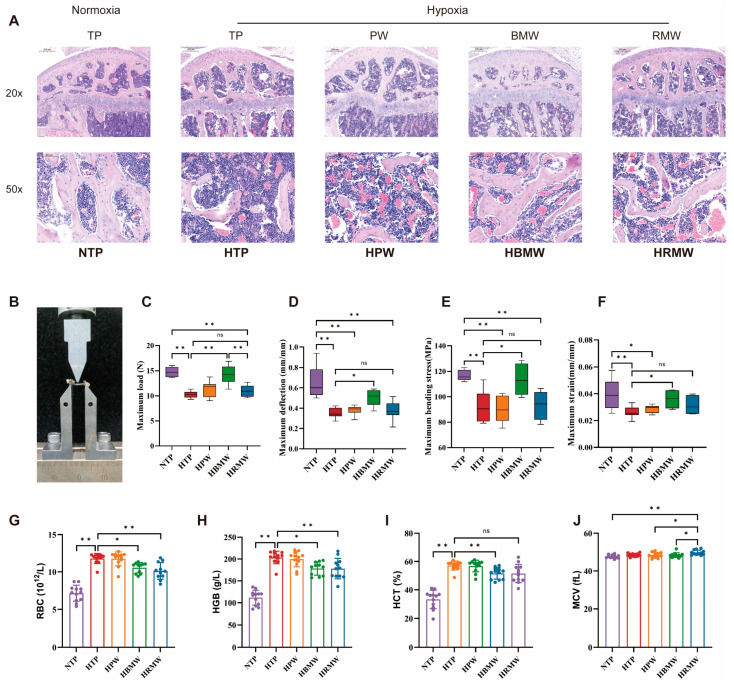
Effects on bone microstructure and related functions. (**A**) Representative full slide images of femora using HE staining. (**B**–**F**) Bone mechanical parameters measured using the three-point bending experiment. (**B**) Schematic diagram of three-point bending experiment. (**C**) Maximum load. (**D**) Maximum deflection. (**E**) Maximum bending stress. (**F**) Maximum strain. (**G**–**J**) Blood routine related to erythroid proliferation, (**G**) RBC, (**H**) HGB, (**I**) HCT, and (**J**) MCV. ns: no significance, * *p* < 0.05, ** *p* < 0.01.

Hematoxylin and Eosin staining was used for bone microstructure analysis (Figure 3A). Compared with the NTP, mice in the HTP group showed fewer and sparser trabeculae in the femur, higher values in bone marrow cavity spaces, and severe microstructural damage to bone tissue. In contrast, the HBMW group exhibited a significant higher value and densely arranged trabeculae, with the microstructure of bone tissue tending to be normalized. The bone structure determines its supportive function, with both its inorganic component (BMD) and organic component (collagen fibers) being crucial for its hardness and toughness. We used a three-point bending test (Figure 3B) to perform biomechanical testing on the femurs of each group. The results show that long-term exposure to high-altitude hypoxia led to lower values in the maximum load, maximum deflection, maximum bending stress, and maximum strain of mouse femurs under stress (Figure 3C–F). However, the HBMW group significantly improved these parameters, enhancing bone hardness and toughness. Additionally, HE staining results reveal a significant higher value in newly formed red blood cells (RBC) in the bone marrow cavity due to hypoxia exposure, suggesting enhanced hematopoiesis, which is possibly related to high-altitude polycythemia (HAPC). We performed blood routine tests (Figure 3G–J) on mice at the end of the experiment after collecting blood from the tail vein. Following high-altitude hypoxic exposure, there was significant proliferation in the erythroid series of mice, characterized mainly by marked higher values in RBC count, hemoglobin (HGB), and hematocrit (HCT). The erythroid proliferation observed in the two mineral water intervention groups (HBMW and HRMW) showed a noticeable improvement, in summary, in addition to improving bone hardness and toughness.

### 3.4. BMW Alters the Gut Microbiota Structure of Mice Under High-Altitude Hypoxia Exposure

After 16S rRNA sequencing of fecal samples collected at the end of the experiment from the NTP, HTP, and HBMW, and following quality control filtering, we obtained a total of 9290 ASVs (Amplicon Sequence Variants), averaging 282 ± 78 ASVs per sample (Supplementary Table S1). As the sequence number increased, the coverage values remained stable, indicating thorough detection in each sample (Supplementary Figure S2A). The Venn diagram shows (Supplementary Figure S2B) that the HBMW group had the most unique feature sequences (220), followed by the HTP group (199), with the NTP having the least (174). The Shannon and Simpson dilution curves (Supplementary Figure S2C,D) show no fluctuation with increasing sample sequencing reads. We then conducted Tukey’s test to assess the differences between the groups. The results from the Shannon and Simpson test (Supplementary Figure S2E,F) indicate that long-term exposure to high-altitude hypoxia changed the structure of gut microbiota in mice. The species richness of the HBMW group and HTP group was significantly higher than that of NTP, while there was no significant difference between the HBMW group and HTP group. The PCoA at the OTU level (Supplementary Figure S2G) reveals distinct clustering patterns among samples from the NTP, HTP, and HBMW groups. The gut microbiota of mice in the HBMW group shows significant differences compared to the other two groups. Samples from different groups of mice exhibit lower similarity in gut microbiota composition, whereas samples within the same group of mice show higher similarity.

**Figure 4:**
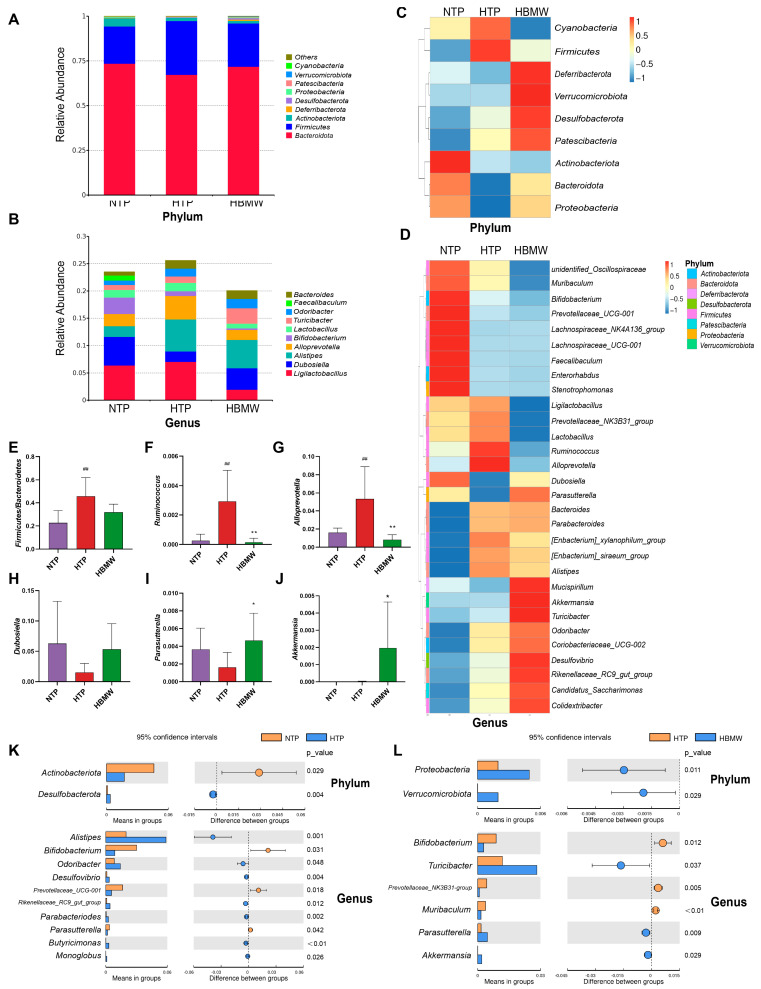
Analysis of gut microbiota structure and differential bacteria in mice. (**A**,**B**) Bar plot of species abundance. (**A**) phylum level and (**B**) genus level (others were not shown). (**C**,**D**) Heatmap of species abundance. (**C**) Phylum level and (**D**) genus level. (**E**–**J**) Abundance of representative differential gut microbiota in the three groups in genus level. (**E**) The ratio of *Firmicutes/Bacteroidetes*, (**F**) *Ruminococcus*, (**G**) *Alloprevotella*, (**H**) *Dubosiella*, (**I**) *Parasutterella*, and (**J**) *Akkermansia*. (**K**,**L**) Analysis of species differences between groups using *t*-test. (**K**) HTP vs. NTP and (**L**) HBMW vs. HTP. * represents HTP vs. HBMW. ^#^ represents NTP vs. HTP. * *p* < 0.05, ** and ^##^ *p* < 0.01.

Using the classify-sklearn algorithm in QIIME2, a pre-trained Naive Bayes classifier was employed to annotate each ASV with species information [33,34]. Based on the species annotation results, the top ten species in each group were selected at the phylum and genus level. Bar plots represent the relative abundance of these species, demonstrating differences in the percentage composition of the gut microbiota in each group. At the phylum level, the top ten bacterial phyla based on species abundance are *Bacteroidota*, *Firmicutes*, *Actinobacteriota*, *Deferribacterota*, *Desulfobacterota*, *Proteobacteria*, *Patescibacteria*, *Verrucomicrobiota*, and *Cyanobacteria* (Figure 4A,C). The top ten species at genus level are, in order, *Ligilactobacillus*, *Dubosiella*, *Alistipes*, *Alloprevotella*, *Bifidobacterium*, *Lactobacillus*, *Turicibacter*, *Odoribacter*, *Faecalibaculum*, and *Bacteroides* (Figure 4B,D). At the genus level, the ratio of *Firmicutes/Bacteroidetes* was significantly higher in the HTP group (Figure 4E). And after long-term natural bicarbonate mineral water intervention, the ratio had a slight tendency to fall back and was not significantly different from the NTP. At the genus level, the HTP group showed significant higher values in the relative abundance of *Ruminococcus* and *Alloprevotella*. Conversely, the relative abundance of *Dubosiella* and *Parasutterella* exhibited lower relative abundances. Following intervention with BMW, these changes in gut microbiota abundance were restored to levels comparable to the normoxia control group (Figure 4F–I). It is noteworthy that the relative abundance of *Akkermansia* showed a significant higher value after the BMW intervention, although the hypoxia did not affect its abundance (Figure 4J). Using a *t*-test to assess differences in microbiota at each taxonomic level, following hypoxic exposure, the HTP group exhibited a significant lower values in *Actinobacteriota* and a high value in *Desulfobacterota* at the phylum level. Following intervention with BMW, there was a notable higher value in the relative abundance of *Proteobacteria* and *Verrucomicrobiota*. At the genus level, the HTP group showed significant higher values in the relative abundance of *Alistipes*, *Odoribacter*, *Desulfovibrio*, *Rikenellaceae_RC9_gut_group*, *Parabacteroides*, *Butyricimonas*, and *Monoglobus*. Conversely, *Bifidobacterium*, *Prevotellaceae_UCG-001*, and *Parasutterella* exhibited lower relative abundances. Following intervention with BMW, *Turicibacter*, *Parasutterella*, and *Akkermansia* showed significantly higher abundances, while *Bifidobacterium*, *Prevotellaceae_NK3B31-group*, and *Muribaculum* showed lower abundances (Figure 4K,L).

### 3.5. BMW Induces Changes in the Metabolite Profiles in Fecal Samples of Mice Under High-Altitude Hypoxia Exposure

The fecal samples at the end of exposure were also subjected to Untargeted Metabolomics. After quality control (QC), a total of 1950 metabolic peaks were detected (pos: 1277; neg: 673). Overall, the quality control analysis showed a Pearson correlation R^2^ > 0.97, close to 1, indicating that the whole detection process had good stability and high data quality. OPLS-DA analysis and 200 permutation tests confirmed the effectiveness of the model. The results show that there were differences in metabolites between the different groups. The slopes of the two slashes are positive, and the intercept of Q2 does not exceed 0.05, suggesting that the model was chosen appropriately and that there was no overfitting. All of the metabolites identified using Untargeted Metabolomic analysis predominantly include lipids and lipid-like molecules, organic acids and derivatives, and organoheterocyclic compounds (Supplementary Figure S3A–D).

**Figure 5:**
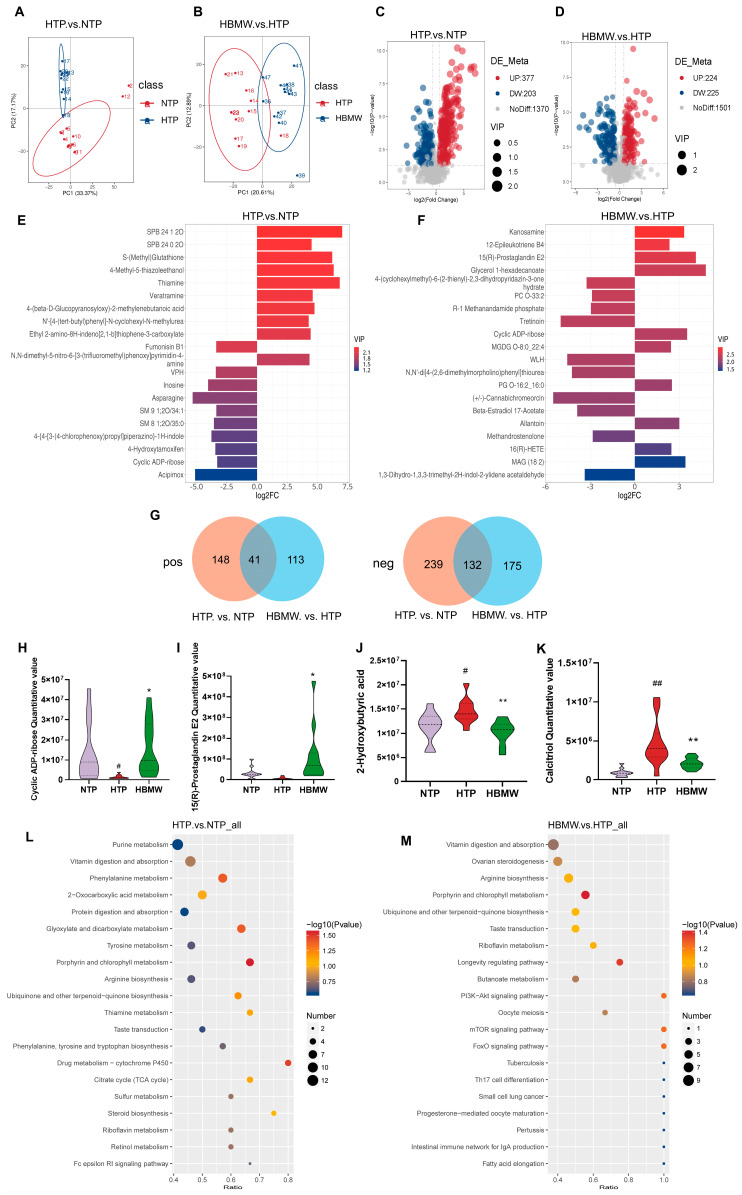
Analysis of differential metabolites and associated differential pathways. (**A**,**B**) PCA to observe the overall distribution trend between the two groups. (**A**) HTP vs. NTP and (**B**) HBMW vs. HTP. (**C**,**D**) Volcano plot showing overall distribution of differential metabolites. (**C**) HTP vs. NTP and (**D**) HBMW vs. HTP. (**E**,**F**) Stem plot of different metabolites. (**E**) HTP vs. NTP and (**F**) HBMW vs. HTP. (**G**) Venn diagram of metabolites between the two comparing groups. (**H**–**K**) Quantitative value of metabolites. (**H**) Cyclic ADP-ribose, (**I**) 15(R)-Prostaglandin E2, (**J**) 2-Hydroxyvaleric acid, and (**K**) Calcitriol. (**L**,**M**) Bubble chart of the enriched KEGG pathway. (**L**) HTP vs. NTP and (**M**) HBMW vs. HTP. * represents HTP vs. HBMW. ^#^ represents NTP vs. HTP. * and ^#^: *p* < 0.05, ** and ^##^: *p* < 0.01.

Using PCA, we observed that the overall distribution of metabolites in the two hypoxic groups was significantly concentrated compared to the NTP ranges in Figure 5A,B. Volcano plots were used to display the overall distribution of these differential metabolites (Figure 5C,D). A comparative metabolic analysis between the NTP and HTP group revealed a total of 377 up-regulated and 203 down-regulated metabolites. Key up-regulated metabolites included S-(Methyl)Glutathione, 4-methyl-5-thiazoleethanol, thiamine, and veratramine, while asparagine, acipimox, inosine, and cyclic ADP-ribose (cADPR) were down-regulated. Comparing the HBMW group with the HTP group, a total of 224 up-regulated metabolites and 225 down-regulated metabolites were identified. Notably, cADPR and prostaglandin E2, which were down-regulated by hypoxia exposure, showed a higher value after BMW intervention (Figure 5E,F). Then, the Venn diagram shows 41 (pos) and 132 (neg) metabolites with significant difference between the groups (Figure 5G). cADPR was lower in value after hypoxia, but recovered after BMW intervention. Similarly, 15(R)-Prostaglandin E2 exhibited a noticeable upward trend after BMW intervention. Conversely, 2-Hydroxyvaleric acid and Calcitriol were high in value after hypoxia exposure, but became lower following BMW intervention (Figure 5H–K). We also analyzed the enriched KEGG pathways annotated for untargeted differentially accumulated metabolites, primarily focusing on metabolism and organismal systems. Pathways such as amino acid metabolism, metabolism of cofactors and vitamins, lipid metabolism, and the digestive and endocrine system were highlighted (Supplementary Figure S3E,F).

Using hypergeometric testing, we identified significant pathways related to differential metabolites (Supplementary Figure S4). Key pathways affected by hypoxia included the porphyrin and chlorophyll metabolism, drug metabolism cytochrome P450, phenylalanine metabolism, and the glyoxylate and dicarboxylate metabolism (HTP vs. NTP). After BMW intervention, the porphyrin and chlorophyll metabolism were significantly down-regulated, and the longevity regulation pathway, FoxO, PI3K-Akt, and mTOR signaling pathways were up-regulated (Figure 5L,M). (All differential metabolites and KEGG pathways are shown in Supplementary Tables S2 and S3).

**Figure 6:**
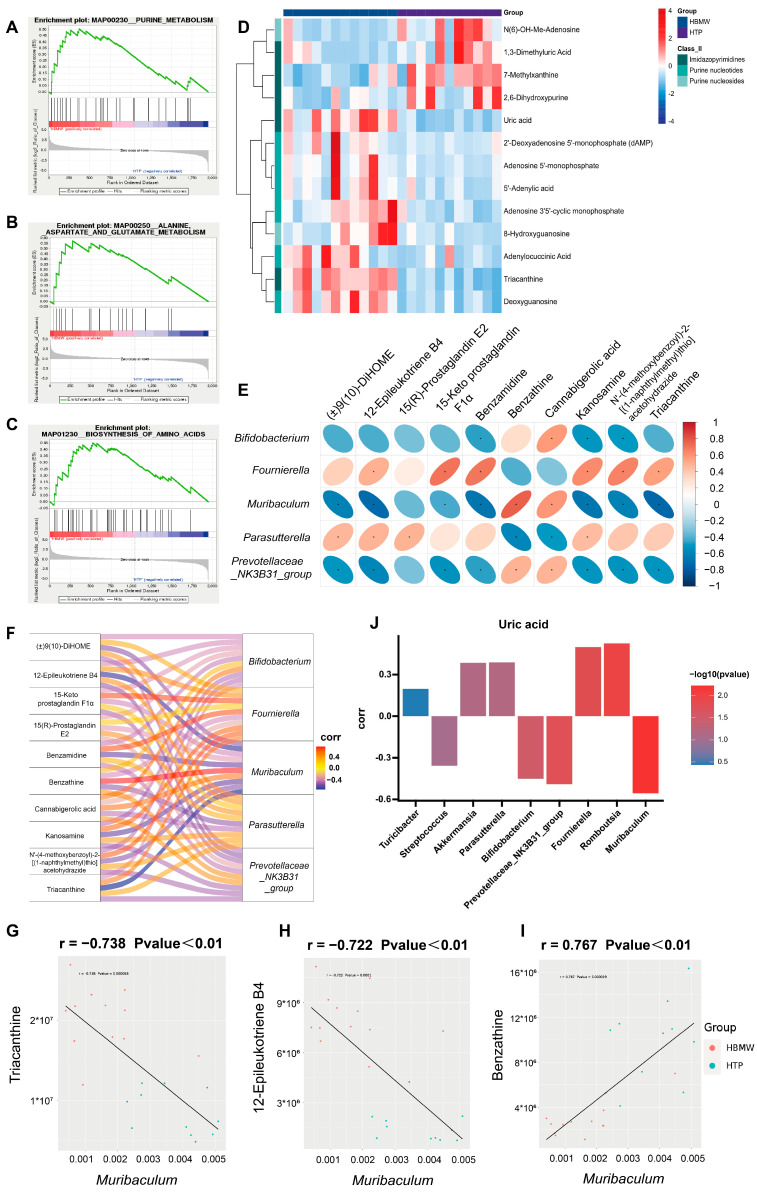
GSEA screens potential pathways and analysis of metabolome–microbe associations after BMW intervention. (**A**–**C**) GSEA of HBMW vs. HTP. (**A**). Purine metabolism. (**B**) Alanine aspartate and glutamate metabolism. (**C**) Biosynthesis of amino acid. (**D**) Heatmap plot of purine metabolites between HBMW and HTP. (**E**) Correlation heatmap plot. (**F**) Correlation Sankey plot. (**G**–**I**) Scatterplot analysis of the correlation between *Muribaculum* and selected metabolites. (**G**) Triacanthine, (**H**) 12-Epileukotriene B4, and (**I**) Benzathine. (**J**) Histogram of the association analysis of uric acid with different microorganisms. The color scale ranges from blue to red in the figure represents the magnitude of the values: blue indicates low values, while red signifies high values. * represent statistical significance, *p* < 0.05. Red indicates high values and blue indicates low values.

Gene Set Enrichment Analysis (GSEA) helps address the limitations of traditional enrichment analysis by providing a more comprehensive understanding of the regulatory roles of functional units. In the comparison between the NTP and HTP groups, we found that pathways, such as aminoacyl-tRNA biosynthesis, biosynthesis of amino acid, and the alanine aspartate and glutamate metabolism, as well as the arginine and proline metabolism, were significantly down-regulated after hypoxic exposure (Supplementary Figure S5). After long-term BMW intervention, the aforementioned amino acid biosynthesis and alanine aspartate and glutamate metabolism showed an upward trend, and purine metabolism pathways were also significantly up-regulated (Figure 6A–C). A heatmap on purine metabolism-related products revealed nine significantly up-regulated products, including uric acid, deoxyadenosine monophosphate (dAMP), and Adenosine 5′-monophosphate, as well as four down-regulated products such as 7-Methylxanthine and 2,6-Dihydroxypurine (Figure 6D).

To evaluate the interaction between gut microbiota and metabolites, we conducted a correlation analysis on significantly different microbiota identified at the genus level and metabolites from Untargeted Metabolomics between the HBMW and HTP groups. The results show significant correlations between most metabolites and gut microbiota. Notably, *Parasutterella* was positively correlated with 15(R)-Prostaglandin E2, and *Fournierella* was positively correlated with 15-Keto Prostaglandin F1α (Figure 6E,F). *Muribaculum* showed a significant negative correlation with Triacanthine and 12-Epileukotriene B4, but a positive correlation with Benzathine (Figure 6G–I).

Therefore, based on the significant differences observed in purine metabolic pathways, we analyzed the microbial correlation with uric acid, the principal product of purine metabolism. Uric acid levels were positively correlated with *Fournierella* and *Romboutsia* and negatively correlated with *Bifidobacterium*, *Prevotellaceae_NK3B31_group*, and *Muribaculum* (Figure 6J).

## 4. Discussion

Water, as an essential substance for survival, typically has a lower mineral content compared to clinical mineral supplements used to treat osteoporosis. However, minerals in water exist in ionic form, making them easier for the body to absorb and providing a stable, continuous, and effective supply [1]. Research suggests that mineral water, particularly the kinds rich in calcium and bicarbonate, could be an ideal substitute for preventing osteoporosis in elderly individuals with chronic acid load [35,36]. Despite this, the effects of BMW on osteoporosis induced by physical factors like high-altitude hypoxia remain unclear.

General conditions (food intake, water intake, and body weight) of mice indicated that exposure to high-altitude hypoxic environments significantly inhibited the mice’s food and water consumption behaviors and caused a decrease in their body weights. Our findings indicate that both types of mineral water increased bone volume fraction and trabecular number while reducing trabecular separation. Notably, only BMW significantly improved the lower BMD caused by high-altitude hypoxia. The HE staining and Micro-CT 3D reconstruction results demonstrate that BMW alleviated hypoxia-induced damage to bone structures. Consequently, biomechanical testing showed a significant increase in bone hardness and strength with BMW supplementation. Furthermore, BMW also mitigated disturbances in calcium–phosphorus metabolism in mice under hypoxic conditions, up-regulated bone formation markers such as BALP, PINP, and BGP, and inhibited osteoclast differentiation. This was also evidenced by a lower in serum bone resorption marker CTX, PTH, and a reduction in the number of osteoclasts in the femur. It is important to note that changes in PTH levels significantly impact the RANKL/OPG ratio, thereby influencing bone resorption [37,38]. In our study, we observed that hypoxic exposure led to a higher value in serum PTH levels in the HTP and lower HBMW. Based on these findings, we hypothesize that BMW may regulate bone resorption at high altitude under hypoxia by modulating PTH levels, which in turn affects the RANKL/OPG ratio. These findings suggest that long-term consumption of BMW may have a preventive effect on high-altitude hypoxia-induced osteoporosis, effectively slowing its progression. In contrast, regular mineral water (RMW) did not produce the same beneficial effects.

High altitude has been shown to affect the composition and diversity of gut microbiota in both humans and animals [39,40,41], and the gut–bone axis has recently garnered attention in the study of bone metabolic disorders. We performed 16S rRNA sequencing to identify differential microbiota at both phylum and genus levels. The results indicate that, following BMW consumption, there was a significant higher value in the relative abundance of *Proteobacteria* and *Verrucomicrobiota* at the phylum level. Notably, the *Firmicutes/Bacteroidetes* ratio, which was higher due to high-altitude hypoxic exposure, exhibited a decreasing trend with BMW administration. *Firmicutes* and *Bacteroidetes* are two dominant groups in the gut microbiota, together comprising approximately 90% of the total microbial community. Changes in this ratio are intricately linked to the host’s health status, influencing aging [42], immune regulation [43,44], and metabolic processes [45,46], and are considered important indicators of gut microbiota health. Hypoxic exposure markedly increased this ratio, while BMW intervention appeared to reverse this trend, suggesting that BMW may alleviate gut microbiota imbalance induced by hypoxia. At the genus level, we observed significant higher values in the abundance of beneficial microbes following BMW consumption such as *Dubosiella*, *Parasutterella*, and *Akkermansia*, while the abundance of others, such as *Ruminococcus* and *Alloprevotella*, showed lower values. Currently, *Ruminococcus* is thought to be positively associated with intestinal inflammation, after an intervention using Bifidobacterium to condition postmenopausal osteoporotic female rats significantly reduces the abundance of *Ruminococcus* [47,48]. In contrast, the relative abundance of *Ruminococcus* and *Alloprevotella* increased after thyroidectomy in a hyperthyroid population [49]. In addition, *Parasutterella* is a previously uncharacterized member of the core gut microbiota and now is proven to be significantly associated with aromatic amino acids, bilirubin, purine, and bile acid metabolism [50]. Notably, *Dubosiella* and *Akkermansia*, identified as dominant probiotics, exhibits various potential benefits for overall host health [51]. *Dubosiella* may be a potential age-related probiotic [52], and *Akkermansia* is described as a paradigm for next-generation beneficial microorganisms which enhances intestinal integrity, modulates insulin resistance, and protects the host from metabolic inflammation [53,54]. Furthermore, research has found that *Akkermansia* is positively correlated with bone formation markers and 25-OH-D and negatively correlated with bone resorption markers [55], underscoring its significant role in maintaining bone homeostasis. Based on these observations, we propose that BMW intervention may operate by modulating the gut microbiota structure, reducing the abundance of inflammation-related bacterial populations and alleviating chronic intestinal inflammation. This, in turn, could help mitigate the bone loss caused by inflammation and relieve osteoporosis. Additionally, it may enhance the body’s metabolic processes and increase the abundance of beneficial gut bacteria, thereby providing a protective effect on the bone metabolism.

Symptoms of osteoporosis are challenging to fully reverse. Greater attention should be focused on therapeutic and metabolic clues [56]. Thus, we employed Untargeted Metabolomics to analyze the preventive effects of BMW on high-altitude hypoxia-induced osteoporosis. Our analysis identified 224 up-regulated metabolites and 225 down-regulated metabolites. Among these, cADPR, which is down-regulated due to hypoxia, exhibited an increasing trend with BMW administration. cADPR primarily regulates calcium ion release in cells and participates in various physiological processes, including cell signaling and immune regulation [57,58]. The activity and function of bone cells are closely related to intracellular calcium ion concentration and its regulation. Therefore, cADPR may indirectly impact the development of osteoporosis by influencing intracellular calcium ion concentrations. Triacanthine is a natural derivative of adenine with good anti-tumor effects [59], but fewer studies have been conducted on its regulating effects of bone metabolism. Prostaglandin E2 (PGE2) secreted by osteoblastic cells activates PGE2 receptor 4 (EP4) in sensory nerves to regulate bone formation by inhibiting sympathetic activity through the central nervous system [60]. Leukotriene B4 and its receptors mediate macrophage migration during the inflammatory process, indicating that it is a potential therapeutic target for inflammatory pathologies [61], which also aligns with the earlier mention of BMW intervention in alleviating intestinal inflammation. Subsequently, we analyzed the KEGG pathways enriched with differential metabolites. Following BMW consumption, we observed significant differences compared to the HTP group in pathways related to the porphyrin and chlorophyll metabolism, longevity regulating pathway, FoxO, and the PI3K-Akt and mTOR signaling pathway. The PI3K/AKT/mTOR (PAM) signaling pathway is a highly conserved network in eukaryotic cells that promotes cell survival, growth, and cell cycle progression [62,63,64]. It is frequently activated in human cancers, and recent studies have highlighted its role in the bone metabolism, potentially involving cellular autophagy [65], anti-inflammation [66], and promotion of osteogenic differentiation [67,68].

Furthermore, GSEA revealed that BMW intervention enhanced amino acid biosynthesis pathways that were down-regulated under hypoxic conditions. Amino acids serve as both an energy source for bones and as structural components, as well as being crucial for bone health [69]. Additionally, we identified significant up-regulation in the purine metabolism pathways. Recent research indicates that disruptions in the purine metabolism are implicated in the occurrence and progression of osteoporosis [70,71]. Our analysis of purine metabolism-related products revealed nine significantly up-regulated metabolites, including uric acid, dAMP, and Adenosine 5′-monophosphate, as well as four down-regulated products such as 7-Methylxanthine and 2,6-Dihydroxypurine. We also conducted a microbiota correlation analysis on uric acid, the primary metabolite of the purine metabolism. The results indicate a positive correlation with *Fournierella* and *Romboutsia*, and a negative correlation with *Bifidobacterium*, *Prevotellaceae_NK3B31_group*, and *Muribaculum*. Uric acid, while being a key metabolite in purine metabolism, has a controversial role in bone metabolism regulation. Some studies have found a positive correlation between serum uric acid levels and BMD [72,73], while others suggested that uric acid accumulation can generate reactive oxygen species (ROS) [74], inhibit antioxidant capacity, induce inflammation, enhance osteoclast bone resorption capability, and ultimately leading to bone loss [75]. Genes necessary for purine degradation are widely present in intestinal bacteria, and studies have shown that fecal transplantation significantly modulates blood uric acid levels [76], indicating that gut microbiota are crucial in maintaining the host’s purine homeostasis. Interventions using BMW in modulating the purine metabolism under high-altitude hypoxia may represent a mechanism through which it supports bone homeostasis via the bone–gut axis. Taken together, BMW may alleviate the progression of high-altitude-induced osteoporosis by altering the structure of gut microbiota and their regulating metabolism.

## 5. Conclusions

High-altitude hypoxic exposure significantly contributes to osteoporosis. However, consuming BMW offers protective benefits against this condition. The results indicate that BMW improves bone health by increasing bone volume, enhancing BMD, and promoting mechanical strength under hypoxic conditions. BMW supplementation effectively regulates calcium and phosphorus metabolism and modulates the biochemical markers associated with bone formation and resorption, thereby slowing the progression of osteoporosis. Furthermore, BMW consumption positively alters the gut microbiota by alleviating structural disturbance caused by hypoxia and increasing beneficial probiotics such as *Akkermansia* and *Dubosiella*, which are linked to improvements in bone health. The results also highlight the potential involvement of signaling pathways such as the PI3K/AKT/mTOR pathway, as well as metabolic processes related to amino acids and purine metabolism in the protective effects of BMW. Overall, our findings present a readily implementable strategy for the prevention of osteoporosis in high-altitude areas, while the mechanisms we identified provide new insights into the etiology and prevention of osteoporosis in high-altitude areas.

## Figures and Tables

**Table 1:** Minerals in different types of drinking water.

	TP	PW	BMW	RMW
pH	7.1	6.3	6.7	6.1
Total dissolved solids (mg/L)	198	50	1626	1967
Ca (mg/L)	39.6	1.6	370.6	376.6
K (mg/L)	1.97	<0.05	13.88	13.54
Na (mg/L)	5.91	0.46	82.96	92.43
Mg (mg/L)	11.2	1.9	89.2	74.9
Mn (mg/L)	<0.01	<0.01	<0.01	0.001
Fe (mg/L)	0.12	<0.01	0.07	0.07
Cu (mg/L)	<0.01	<0.01	<0.01	0.01
Zn (mg/L)	<0.01	<0.01	<0.01	0.01
Li (mg/L)	0.036	0.033	0.216	0.216
Sr (mg/L)	0.14	0.03	1.31	1.31
F^−^ (mg/L)	0.107	<0.1	<0.1	<0.1
Cl^−^ (mg/L)	16.5	0.37	45	42.5
SiO_3_^2−^ (mg/L)	1.19	<0.1	22.42	20.36
SO_4_^2−^ (mg/L)	21.6	0.9	54	472
CO_3_^2−^ (mg/L)	N.D.	N.D.	3.0	1.2
HCO_3_^−^ (mg/L)	18.65	6.36	1651.5	335.6

N.D.—not detected.

## Data Availability

The original contributions presented in this study are included in the article/Supplementary Materials. Further inquiries can be directed to the corresponding author.

## Supplementary Materials

The following supporting information can be downloaded at: https://www.mdpi.com/article/10.3390/nu17060998/s1, Figure S1. Determination of the index of bone density observation in mice on day 0 by DXA. (A) Overall BMD in Day 0, (B) femoral BMD in Day 0. Figure S2. Sequencing analysis, richness and diversity of gut microbiota in mice. (A) Good_coverage dilution curve, (B) Wayne diagram analysis is carried out according to OTU abundance information of different groups, (C,D) Shannon dilution curve and index differential analysis, (E,F) Simpson dilution curve and index differential analysis, (G) PCoA principal coordinate analysis. **, *p* < 0.01. Figure S3. Data quality control, classification of metabolites and taxonomic annotation of untargeted metabolomics analysis. (A) Correlation analysis of QC samples. (B) OPLS-DA score scatter plot. (C) Permutation test of OPLS-DA. (D) Pie chart of classification of metabolites. (E,F) KEGG classification analysis. g HTP vs. NTP; h HBMW vs. HTP. Figure S4. Hypergeometric test of metabolites after annotation of KEGG information. N represents the number of metabolites with KEGG annotation information among all metabolites, n represents the number of differential metabolites in N, M is the number of metabolites annotated to a particular KEGG entry in all. x is the number of differential metabolites annotated to a particular KEGG entry, and *p*-values were calculated to screen for KEGG pathways significantly enriched in differential metabolites at a threshold of *p* < 0.05. Figure S5. Gene Set Enrichment Analysis(GSEA) of HTP. vs. NTP. (A) Aminoacyl-tRNA biosynthesis, (B) Biosynthesis of amino acid, (C) Alanine aspartate and glutamate metabolism, (D) Arginine and proline metabolism. Table S1. Results obtained from the sequencing data after quality control and noise reduction processes. Table S2. Results of quantification of all identified metabolites. Table S3. KEGG Pathway enrichment of differential metabolites.

## Abbreviations

The following abbreviations are used in this manuscript:

DXA	Dual-energy X-ray Absorptiometry
UHPLC-MS/MS	Ultra-High Performance Liquid Chromatography coupled with Tandem Mass Spectrometry
PAM	PI3K/AKT/mTOR
BMD	Bone Mineral Density
MSCs	Mesenchymal Stem Cells
SPF	Specific Pathogen-Free
BV/TV	bone volume fraction
Tb.Th	trabecular thickness
Tb.N	trabecular number
Tb.Sp	trabecular bone spacing
SMI	structure model index
EUTM	Electronic Universal Testing Machine
Fmax	maximum load
dmax	maximum deflection
CTX I	Cross Linked C-Telopeptide of Type I Collagen
BALP	Bone Alkaline Phosphatase
PINP	Propeptide of Type I Procollagen
PTH	Parathyroid Hormone
BGP	Bone Gla-protein
CT	Calcitonin
CTAB	Cetyltrimethylammonium Ammonium Bromide
TRAP	Tartrate-resistant Acid Phosphatase
HAPC	High-altitude Polycythemia
cADPR	cyclic ADP-ribose
GSEA	Gene Set Enrichment Analysis
dAMP	deoxyadenosine monophosphate
PGE2	Prostaglandin E2
EP4	Prostaglandin E2 receptor 4
