# Supplementary material for: Bicarbonate-Rich Mineral Water Mitigates Hypoxia-Induced Osteoporosis in Mice via Gut Microbiota and Metabolic Pathway Regulation
Source: Nutrients. 2025 Mar 12;17(6):998. doi: 10.3390/nu17060998 (PMC11944587; doi:10.3390/nu17060998)
Supplement: Supplementary file 1 [file nutrients-17-00998-s001.zip › Supplementary Figures.pdf]

Supplementary Figures:

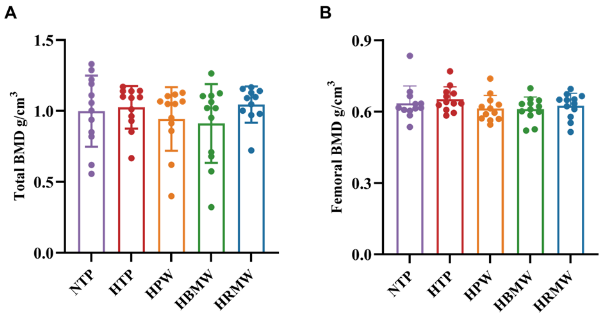

Supplementary Figure S1. Determination of the index of bone density observation in mice on day 0 by DXA. (**A**) Overall BMD in Day 0, (**B**) femoral BMD in Day 0.

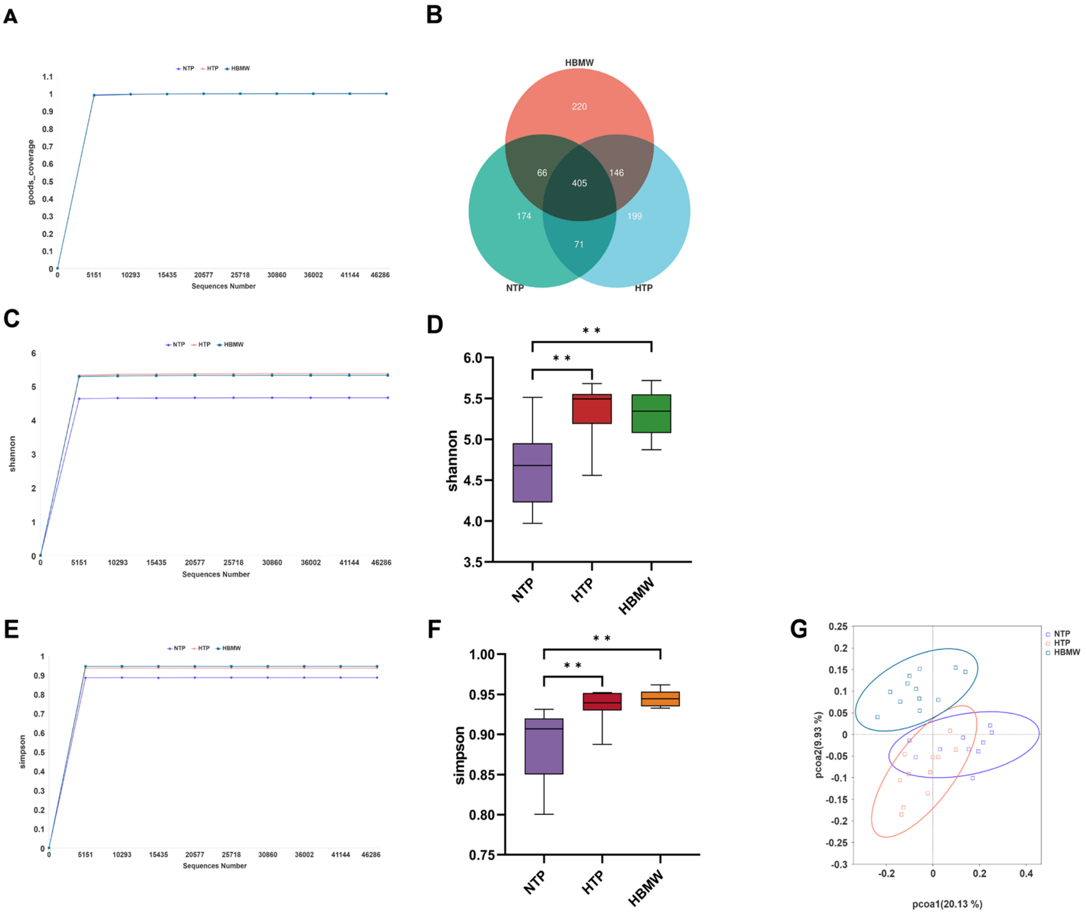

Supplementary Figure S2. Sequencing analysis, richness and diversity of gut microbiota in mice. (**A**). Good_coverage dilution curve, (**B**). Wayne diagram analysis is carried out according to OTU abundance information of different groups, (**C**,**D**). Shannon dilution curve and index differential analysis, (**E**,**F**). Simpson dilution curve and index differential analysis, (**G**). PCoA principal coordinate analysis.**, $p < 0.01$.

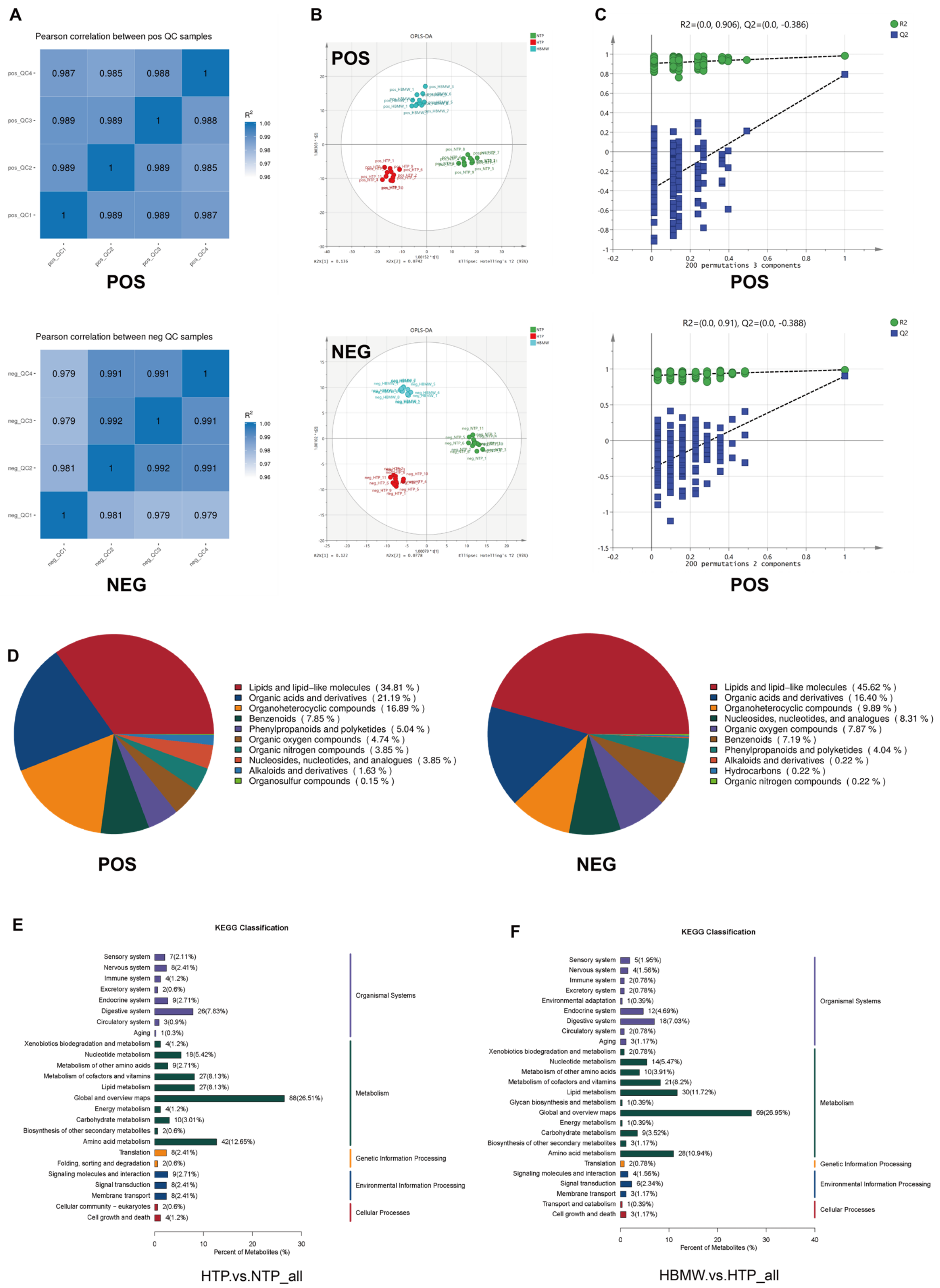

Supplementary Figure S3. Data quality control, classification of metabolites and taxonomic annotation of untargeted metabolomics analysis. (**A**) Correlation analysis of QC samples. (**B**) OPLS-DA score scatter plot. (**C**) Permutation test of OPLS-DA. (**D**) Pie chart of classification of metabolites. (**E**,**F**) KEGG classification analysis. g HTP vs. NTP; h HBMW vs. HTP.

$$p\_value = 1 - \sum_{j=0}^{x-1} \frac{\binom{M}{j}\binom{N-M}{n-j}}{\binom{N}{n}}$$

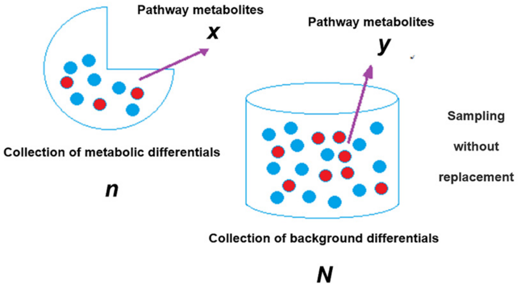

Supplementary Figure S4. Hypergeometric test of metabolites after annotation of KEGG information. N represents the number of metabolites with KEGG annotation information among all metabolites, n represents the number of differential metabolites in N, M is the number of metabolites annotated to a particular KEGG entry in all. x is the number of differential metabolites annotated to a particular KEGG entry, and p-values were calculated to screen for KEGG pathways significantly enriched in differential metabolites at a threshold of $p < 0.05$.

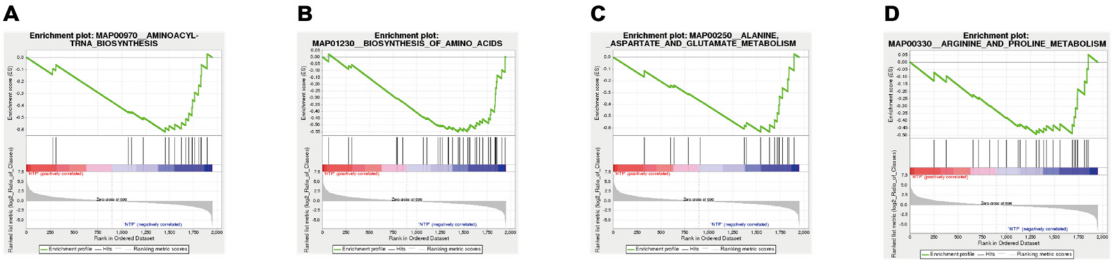

Supplementary Figure S5. Gene Set Enrichment Analysis(GSEA) of HTP. vs. NTP. (**A**) Aminoacyl-tRNA biosynthesis, (**B**) Biosynthesis of amino acid, (**C**) Alanine aspartate and glutamate metabolism, (**D**) Arginine and proline metabolism.
